# Face morphing attacks: Investigating detection with humans and computers

**DOI:** 10.1186/s41235-019-0181-4

**Published:** 2019-07-29

**Authors:** Robin S. S. Kramer, Michael O. Mireku, Tessa R. Flack, Kay L. Ritchie

**Affiliations:** School of Psychology, University of Lincoln, Lincoln, LN6 7TS UK

**Keywords:** Morphing attack, Face morph, Fraud, Face matching, Morph detection

## Abstract

**Background:**

In recent years, fraudsters have begun to use readily accessible digital manipulation techniques in order to carry out face morphing attacks. By submitting a morph image (a 50/50 average of two people’s faces) for inclusion in an official document such as a passport, it might be possible that both people sufficiently resemble the morph that they are each able to use the resulting genuine ID document. Limited research with low-quality morphs has shown that human detection rates were poor but that training methods can improve performance. Here, we investigate human and computer performance with high-quality morphs, comparable with those expected to be used by criminals.

**Results:**

Over four experiments, we found that people were highly error-prone when detecting morphs and that training did not produce improvements. In a live matching task, morphs were accepted at levels suggesting they represent a significant concern for security agencies and detection was again error-prone. Finally, we found that a simple computer model outperformed our human participants.

**Conclusions:**

Taken together, these results reinforce the idea that advanced computational techniques could prove more reliable than training people when fighting these types of morphing attacks. Our findings have important implications for security authorities worldwide.

**Electronic supplementary material:**

The online version of this article (10.1186/s41235-019-0181-4) contains supplementary material, which is available to authorized users.

## Significance

In order to minimize the use of fraudulent documents as forms of identification, anti-counterfeit measures such as watermarks are often included. With an increase in the detection of fraudulent IDs, security officers have recently seen a rise in the use of fraudulently obtained genuine (FOG) documents. As the name suggests, these involve deception during the application process in order to obtain a genuine document, equipped with all the necessary watermarks, and so on. One method used by fraudsters is to submit a morph image (a 50/50 average of two people’s faces) for inclusion in an official document like a passport. If both people sufficiently resemble the morph, they could both use the resulting genuine passport for international travel. Recent research has begun to investigate whether people can detect morphs and has suggested that training might provide an effective way to increase performance. Here, we reconsidered these findings with the use of higher-quality morphs, where every effort was made to produce images comparable with those we expect criminals to use. We found that on-screen morph detection was poor and training did not lead to improvements. When morphs were compared to faces during a live interaction, they were accepted at concerning levels and, again, detection was error-prone. Importantly, we found that a simple computer model performed better than our human participants, suggesting that security agencies should focus on automated solutions rather than training people when fighting morphing attacks.

## Background

The use of biometrics in identification is commonplace across a variety of contexts. For example, face photographs are featured in many forms of documentation internationally, including passports and driving licenses. Our reliance on the face as a means of identification is likely a result of our belief that we are face experts. However, in reality, we are only *familiar* face experts (Young & Burton, 2018). Numerous studies have now shown that we are error-prone when making decisions based upon unfamiliar faces (e.g. Bruce, Henderson, Newman, & Burton, 2001; Burton, White, & McNeill, 2010; Jenkins, White, Van Montfort, & Burton, 2011; Kemp, Towell, & Pike, 1997). Further, and perhaps surprisingly, trained passport officers perform at similar levels to untrained university students (White, Kemp, Jenkins, Matheson, & Burton, 2014).

Errors with unfamiliar faces become especially problematic when dealing with various types of fraudulent identification. For instance, researchers in recent years have begun to investigate the issue of “face morphing attacks” (Ferrara, Franco, & Maltoni, 2014). This term refers to the following three-step process to obtain a passport fraudulently. Person A (who has no criminal record) creates a morphed photo of himself and person B (whose prior record prevents him from international travel). First, person A submits this AB morph as his ID photograph with his passport application. Second, the morph is compared with previous images of person A that are kept on file and the application is subsequently approved by the passport issuing officer on the grounds that the image sufficiently resembles him. Third, person A gives this FOG (Interpol, n.d.) passport to person B, who then proceeds to use it during travel as he also resembles the morph image sufficiently to pass through border control.

Problematically, since the document itself is genuine, typical anti-counterfeit measures (e.g. the use of security watermarks, inks, and fibers) are powerless to detect these types of fraud. Therefore, detection must rely upon comparing the morph with previously stored face photographs (at the point of issuance) or the “live” face (at the point of presentation for travel). As digital image manipulation software becomes more advanced, the resulting morphs become more difficult to detect. One approach is to develop increasingly sophisticated computer methods for morph detection (e.g. Makrushin, Neubert, & Dittmann, 2017; Neubert, 2017; Raghavendra, Raja, Venkatesh, & Busch, 2017a, 2017b; Scherhag, Nautsch, et al., 2017; Scherhag, Raghavendra, et al., 2017; Seibold, Samek, Hilsmann, & Eisert, 2017, 2018). For example, inconsistencies between the reflections visible in the eyes and skin could signal a morphed image (Seibold, Hilsmann, & Eisert, 2018). Such techniques may be incorporated into automated border control (ABC) systems in order to prevent the use of morph images.

In many situations, however, the decision to accept an ID image is left to a human operator. Indeed, even in face matching scenarios where algorithms are initially employed, human users are often presented with a “candidate list” and are required to make the final selection, potentially reducing the overall accuracy of the process (White, Dunn, Schmid, & Kemp, 2015). Although important across a variety of contexts, the question of whether people are able to detect morphs and/or whether they accept such images as genuine ID photographs has received little attention to date.

Ferrara, Franco, and Maltoni (2016) provided evidence that several computer algorithms performed with high error rates when tasked with detecting morph images. In addition, they found that human performance on their task was also poor, with morphs going undetected in most cases (see Makrushin et al., 2017, for similar findings). In line with previous work on face matching with expert populations (White et al., 2014), their results also showed that professionals working in the field (border guards) were no better than university students and employees in detecting morphs.

Recently, two articles by Robertson and colleagues have specifically focused on human performance in the matching and detection of morphs. In the first, participants completed computer tasks in which they decided whether two face images onscreen depicted the same person or not (Robertson, Kramer, & Burton, 2017). In seven trials, the two images were different photographs of the same face, and in another seven trials, the images were photographs of two different people. For the remaining 35 trials, a face photograph was paired with a morph containing differing amounts of that face and a second person. (When creating morphs, the researcher can specify the percentage weighting of each identity contained in the final image.) The results demonstrated that 50/50 morphs (weighting both identities equally) were accepted as “matches” for the faces they were paired with on 68% of trials. After providing instructions regarding the nature of morphs, and with the additional response option of “morphed image,” participants subsequently accepted them as “matches” on only 21% of trials. Taken together, the authors suggested that erroneously accepting morphs as ID images was common, but these errors can be significantly reduced through instruction.

In the second article, the researchers investigated whether people were able to detect morph images and whether training could help with this task (Robertson et al., 2018). Participants were shown ten-image arrays containing a mixture of the morph images and exemplars (original, unmorphed faces) used in the previous article and were asked to identify which were the morphs. Performance was poor, with the 50/50 morphs resulting in average *d’* sensitivities of 0.56 and 0.96 (for the two groups that took part: training versus none), suggesting that morphs were not readily detected. However, providing information regarding the nature of the morphs, along with some tips to help with identifying them, resulted in a significant increase in sensitivity (to 2.69 and 2.32, respectively). An additional training protocol, in which feedback was provided via a two-alternative forced choice (2AFC) task, also led to a further benefit for the group that received it (the first mentioned in the values reported above). The authors concluded that people were poor at detecting morphs, but that training could significantly improve performance.

**Fig. 1:**
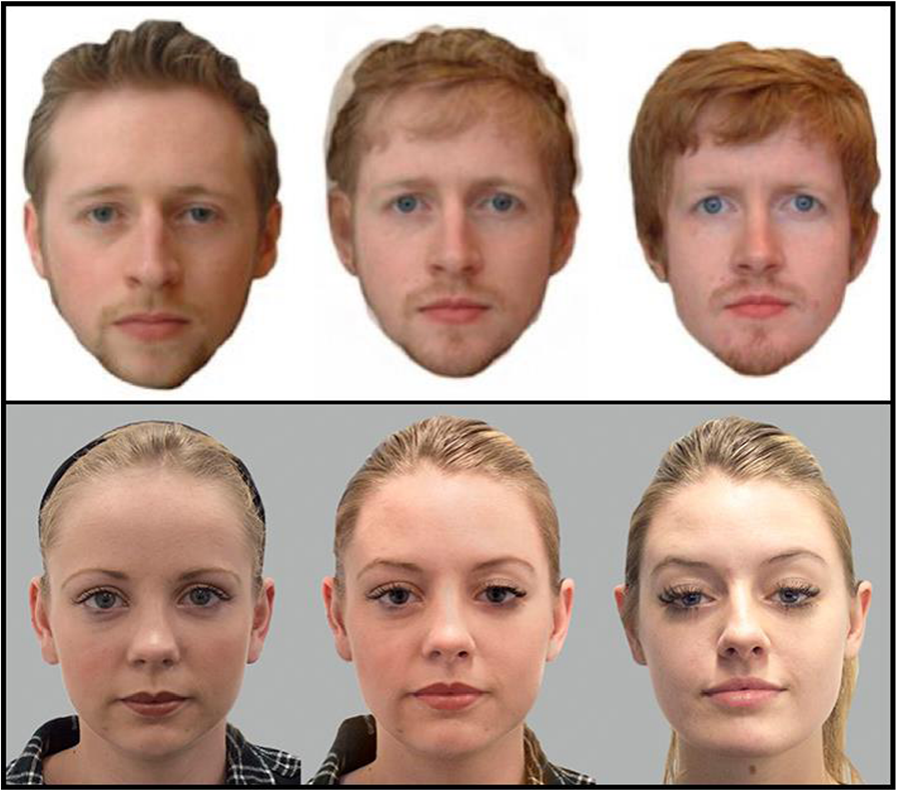
*Top:* An example of the images used in previous work (adapted from Robertson et al., 2018). *Bottom:* An example of the images used in the current work (Experiment 3^1^). The three faces depict two individuals (*left*, *right*) and a morph created using these images (*center*). The individuals pictured have given permission for their images to be reproduced here

As mentioned earlier, with improvements in image manipulation techniques, and in combination with a criminal’s determination to avoid being caught, we should expect that real-world morphs will be made with a level of sophistication that renders them virtually undetectable to the human eye. Problematically for the two articles investigating human acceptance and detection of morphs (Robertson et al., 2017, 2018), the images used were not representative of the level of cutting-edge methods that are likely to be applied by fraudsters. Although the initial face averaging was carried out using advanced morphing software (JPsychomorph; e.g. Benson & Perrett, 1993), there was no subsequent “touch up” stage in order to remove artefacts that are known to result from the averaging process (e.g. the presence of a secondary outline for the hair). As Fig. 1 (top row) illustrates, the 50/50 morph (center) included obvious artefacts that can be easily removed using image-editing software. Indeed, these artefacts were highlighted to participants during the morph detection training phase of both previous studies: “look for a ‘ghost-like’ outline of another face; look for the outline of another person’s hair over the forehead” (Robertson et al., 2018, p. 4). In addition, by presenting faces that have been cropped to remove the neck and background, these images did not conform to real-world ID specifications and also highlighted to participants that all the images had been altered to some extent. For these reasons, we predict that the performance levels reported, along with the apparent training benefits, may only be of limited utility with regard to real-world behaviors when using more realistic images.

In the current set of studies, we aim to address these issues by creating higher-quality morph images and investigating both human and computer detection of these images. It is important to determine whether people accept morphs, or can detect their use, when every effort is made to produce images that reflect real-world fraud. For example, if training methods were implemented with the assumption that morph detection would be significantly improved, this might result in a false sense of security (literally) for passport control and issuing officers. Therefore, in this paper, we investigate human morph detection performance with and without training, reflecting a passport-issuing context (Experiments 1 and 2), whether people accept morphs as ID images in a “live” task, reflecting a border control scenario (Experiment 3), and, finally, whether computational modelling outperforms human detection, providing a more suitable alternative than training people (Experiment 4).

## Experiment 1: Replication of Robertson et al. (2018) using higher-quality morphs

In the first experiment, we repeated the design and procedure of Robertson et al. (2018) with our higher-quality morphed images. In addition, we determined whether the previously implemented training paradigm (forced choice trials with feedback) would result in improved morph detection performance with our more sophisticated morphs.

## Method

### Participants

Eighty participants (57 women; age *M* = 21.2 years, *SD* = 7.2 years; all self-reported as White or White-mixed ethnicities) were recruited for this experiment, based on an opportunity sample of friends, family, and through approaching students and staff on campus.

The University’s School of Psychology Research Ethics Committee approved all the experiments presented here (approval code PSY171881) which were carried out in accordance with the provisions of the World Medical Association Declaration of Helsinki. In all experiments, participants provided written informed consent before taking part.

### Materials

Digital face photographs were taken from two different databases used in previous research (Set 1: *n* = 262; Set 2: *n* = 224; Kramer, Jones, & Ward, 2012; Scott, Jones, Kramer, & Ward, 2015; Scott, Kramer, Jones, & Ward, 2013). To create morphs for our pre- and post-training morph detection tasks, we paired 120 individuals from our Set 1 database to create 60 morphs (43 female). Our 60 exemplar (non-morphed) images (also taken from Set 1) all showed new identities not used in the creation of the morphs. In line with Robertson et al. (2017, 2018), we used JPsychomorph to create our morph images, although crucially, we then modified these images using Adobe Photoshop in order to remove any noticeable artefacts of the averaging process. For further details of the creation of the images, see Additional file 1.

To create morphs for our morph training task, we paired 40 individuals from our Set 2 database and then created 20 morphs (12 female) using the same procedure as above (see Additional file 1). Similarly, we selected 20 individuals (12 female) from the same set for our exemplar images and processed the images as before. Again, these individuals had not appeared in any of the morph pairings.

All images were resized to match the dimensions of official UK passport photographs (3.5 × 4.5 cm).

### Procedure

All details of the procedure were identical to that of Robertson et al. (2018) unless otherwise specified, although we excluded their final face matching test as this was not central to our question. Here, participants completed a pre-training morph detection task, a morph training or control task, and then a post-training morph detection task.

In the pre-training baseline morph detection task, participants were asked to identify morph images in ten-image arrays, providing an initial measure of ability to detect morphs before any training or guidance had been given. On each of the six trials, participants were shown ten faces (five morphs, five exemplars), along with the question, “Which of these images are face morphs (a blend of two faces)?”,^2^ with the option of entering between zero and all ten faces.^3^ The on-screen locations of the morphs and exemplars were randomized for each trial; the 60 faces (30 morphs, 30 individuals) were randomly selected from the set of 120 images described above.

Next, as in Robertson et al. (2018), participants viewed a set of “morph fraud/detection tips” onscreen (“tips screen”). This provided an example of two exemplar photographs, along with the resulting morph created by averaging those two images together (featuring two women who did not appear in any of the experimental trials, either individually or as a morph.) The tips screen also suggested two possible ways to identify morphs: (1) “morphs often have smoother skin than normal photographs”; and (2) “morphs may also show irregularities within the hair’s texture.” These suggestions were tailored to our specific morph set and therefore differed from those provided by Robertson et al. (2018), who recommended that participants look for artefacts that we had digitally removed (see Fig. 1 bottom row). Importantly, in line with Robertson et al., participants in both groups (morph training, control) were shown these tips.

Following the tips screen, participants completed either a morph training task (*n* = 40) or a control task (*n* = 40), with alternating assignment to these tasks. In the morph training task (20 trials), pairs of faces were presented onscreen (one morph, one exemplar) and participants were asked to select the morphed face photo. In the control task (20 trials), pairs of letter circles were presented onscreen (one containing only letters, the other also containing one number) and participants were asked to identify which circle contained the number. Left/right locations for the morphs and letter circles were counterbalanced across trials. Both tasks provided feedback regarding the correct answer before the next trial appeared.

Finally, after completing either the morph training or control task, participants were given a post-training morph detection task. This was identical to the pre-training version (see above) except that a different set of 60 faces (the remaining faces from our set of 120) appeared in the arrays.

## Results

In order to determine whether participation in the morph training task resulted in improved performance when comparing the pre- and post-training blocks, percentage correct was analyzed using a 2 (Group: morph training vs control) × 2 (Session: pre-training vs post-training) mixed analysis of variance (ANOVA). Group varied between participants while Session varied within participants. Overall, performance on the morph detection task was poor. We found a significant main effect of Group, *F*(1, 78) = 6.32, *p* = 0.014, *η*^2^_p_ = 0.75, with the control group (*M* = 56.0%) performing better than the training group (*M* = 52.1%). However, since assignment of participants to these groups was essentially random, any difference between groups can only be explained by chance. Indeed, this group difference translates into the control group scoring an average of only one more correct response on the task than the training group. More importantly for the current work, we found no main effect of Session, *F*(1, 78) = 0.05, *p* = 0.829, *η*^2^_p_ = 0.00, and no significant Group × Session interaction, *F*(1, 78) = 0.00, *p* = 0.971, *η*^2^_p_ = 0.00.

**Fig. 2:**
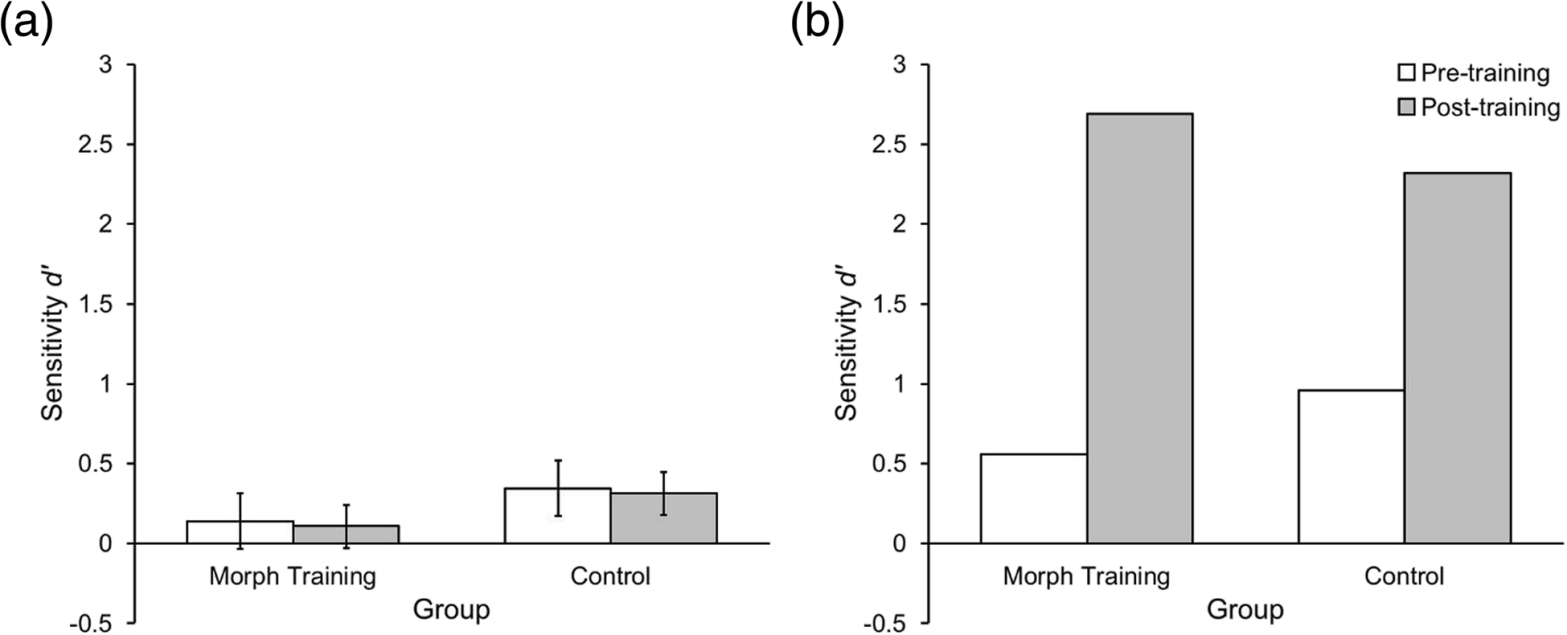
A comparison of Experiment 1 and previous work. A plot of sensitivity data from (**a**) the current experiment and (**b**) Robertson et al. (2018), displayed on the same axes. Data in (**b**) display the results of Robertson’s 50% morphs only. *Error bars* represent 95% confidence intervals

Robertson et al. (2018) analyzed their results using signal detection measures. Therefore, in order to directly compare our data with theirs, we also carried out these analyses. Our ANOVA of *d’* sensitivity showed the same pattern of results as the percentage correct analysis reported above (see Fig. 2 and Additional file 1).

Finally, in line with Robertson et al. (2018), we investigated performance during the morph training and control tasks. For the morph training task, percentage correct over the 20 trials (*M* = 51.3%) was not significantly different from chance (50%) performance, *t*(39) = 0.72, *p* = 0.475, Cohen’s *d* = 0.11. In addition, percentage correct on the first five (*M* = 54.0%) and last five trials (*M* = 47.0%) did not significantly differ from each other, *t*(39) = 1.51, *p* = 0.137, Cohen’s *d* = 0.24, suggesting no improvement as the training task progressed. In contrast, analysis of the control task showed that accuracy was at ceiling (*M* = 99.4%), which was expected, given the simplicity of the task.

## Discussion

The results of this experiment demonstrated two important findings. First, the ability to detect morphs was poor on this task. Sensitivity was only slightly above zero (and not different from zero in some cases). Second, providing training did not lead to an improvement. Indeed, accuracy during training was at chance levels and failed to improve over the 20 trials, despite the feedback that was given after each response.

Previous research (Robertson et al., 2018) showed poor performance on the baseline morph detection task (although at levels higher than in the current experiment) and substantial improvements after receiving detection tips and training. As Fig. 2 illustrates, our current results and those of Robertson et al. (2018) are strikingly different when displayed on the same axes. It is clear that our morphs were more difficult to detect; even when we provided a tailored set of tips (specifically updated to address characteristics that may reveal flaws in our images), no performance increase was seen.

In addition, morph detection during our training task differed substantially from detection rates for the same task in Robertson et al. (2018). Where Robertson and colleagues found that morphs were correctly chosen in the 2AFC training task in 89% of trials, we showed chance-level morph detection (51%). This further highlights that our high-quality morph faces were difficult to detect.

It is, however, unclear as to what should be considered chance-level detection in the pre- and post-training morph detection tasks, where perfect performance would be selecting all five morphs from each ten-image array. Therefore, we designed Experiment 2 in order to establish detection levels for our morphs in a task where chance was 50%.

## Experiment 2: Forced choice morph detection and tips

Here, we explored morph detection using a forced choice paradigm. By presenting a single image on each trial, with participants deciding whether the image is a morph or not, we have a clear notion of chance performance (i.e. 50%) and there are no other images on screen with which to simultaneously compare the image in question (unlike Experiment 1). In addition, making decisions based on single images more closely parallels the real-world task of morph detection that passport officers and other professionals carry out each day. There is no obvious situation in which a large array, containing both morphs and individuals’ images, would be presented for consideration.

Finally, we used a more limited “training” design in that half of the participants viewed the morph detection tips screen before the detection task. We chose not to include the full training task (utilizing feedback) featured in Experiment 1, given that our results provided no evidence that this method of training resulted in improved performance.

## Method

### Participants

Forty participants (21 women, age *M* = 23.9 years, *SD* = 10.8 years; all self-reported as White ethnicity) were recruited for this experiment in the same manner as Experiment 1. There was no overlap between this sample of participants and those who took part in Experiment 1.

### Materials

The 120 images used in the morph detection task in Experiment 1 were used here.

### Procedure

Participants completed a forced choice morph detection task, either viewing the tips screen beforehand (*n* = 20) or not (*n* = 20). Assignment to these groups (tips screen vs control) alternated as above, with odd numbered participants viewing the tips screen and even numbered participants not. The tips screen was identical to the one used in Experiment 1, providing information about morphs, a visual example of their creation, and two suggestions for identifying morphed images.

For the task itself, from the 120 images created using our Set 1 database (60 morphs and 60 exemplars), each participant was presented with a randomly selected 30 morphs and 30 exemplars. The order of presentation of these faces was also randomized. On each trial, one face appeared onscreen, along with the question, “Is this image a face morph (a blend of two faces)?” Participants selected either “yes” or “no” onscreen using the mouse. No feedback was given at any point during the task.

## Results

As Table 1 illustrates, performance on the forced choice morph detection task was poor. In order to determine whether viewing the tips screen before completing the task resulted in improved performance, percentage correct was analyzed using an independent samples *t*-test. We found no significant difference between the two groups, *t*(38) = 1.04, *p* = 0.304, Cohen’s *d* = 0.33.

**Table 1:** A summary of the data for Experiment 2

Group	Percentage correct, %	Hits	False alarms	*d’*	*c*
Tips screen	57.1 [52.4, 61.7]	0.59 [0.53, 0.64]	0.45 [0.38, 0.51]	0.38 [0.12, 0.64]	−0.04 [− 0.14, 0.06]
Control	53.7 [48.6, 58.7]	0.50 [0.42, 0.58]	0.43 [0.34, 0.51]	0.22 [−0.07, 0.50]	0.11 [−0.08, 0.29]

*Note.* Values represent the means, with 95% confidence intervals shown in square brackets

We also analyzed signal detection measures using the following definitions: *Hit* – the image was a morph and participants responded “morph”; and *False alarm* – the image was an individual and participants responded “morph.” For both *d’*, *t*(38) = 0.90, *p* = 0.374, Cohen’s *d* = 0.28, and *c*, *t*(38) = 1.47, *p* = 0.150, Cohen’s *d* = 0.46, we again found no significant difference between the two groups. That the latter comparison produced a near-medium effect size perhaps provided some evidence that participants who viewed the tips screen showed a criterion shift towards responding “morph” more often when completing the task.

Finally, we compared performance levels to those predicted by simple guessing. For percentage correct, the training group performed significantly better than chance, *t*(19) = 3.19, *p* = 0.005, Cohen’s *d* = 0.71, but the control group did not, *t*(19) = 1.52, *p* = 0.145, Cohen’s *d* = 0.34. This pattern was mirrored in the analysis of *d’*, where the training group’s sensitivity was significantly above zero, *t*(19) = 3.11, *p* = 0.006, Cohen’s *d* = 0.70, but the control group’s sensitivity was not, *t*(19) = 1.61, *p* = 0.124, Cohen’s *d* = 0.36.

## Discussion

The results of this experiment confirmed that morph detection was poor, using a task where chance levels were easily defined and the forced choice procedure better reflected real-world decision-making. That *d’* sensitivities were very similar to the levels found in Experiment 1 provided additional evidence of the difficulties that participants had with detecting morphs across multiple paradigms.

Although Experiment 1 demonstrated that neither the tips screen nor the morph training led to an improvement in performance, the current experiment produced more ambiguous results. We found that viewing the tips screen did not significantly increase morph detection abilities, but those participants did perform at above chance levels. In comparison, responses given by participants in the control group did not differ from those expected by simple guessing.

Taken together, the results of the two experiments might suggest that the tips screen has the potential to increase morph detection levels. However, this benefit may be counteracted by: (1) carrying out another task (feedback training or an irrelevant letters task) before morph detection is measured for the second time; and/or (2) the use of a ten-image array paradigm, incorporating additional noise in the data due to the uncertainty as to how many images should be selected on each trial. Whether viewing a tips screen improved detection or not, we can say with certainty that any increase in performance was small and detection levels both with and without training/tips were low.

Both Experiments 1 and 2 addressed the first process involved in using a FOG passport – when issuing a passport, personnel must compare the newly submitted morph image to the previous passport image of one of the identities. Our results have shown that it is unlikely that a morph image would be detected at this stage and that training would not help in this process. The next experiment addresses the second stage of FOG passport criminality – its usage. In this situation, a border control officer or other official must compare a live person to the morph image.

## Experiment 3: Live face matching

Experiments 1 and 2 demonstrated that people were poor at identifying our face morphs and that the training methods we explored did not result in significantly improved performance. If this type of image cannot be easily distinguished from standard exemplar images, then this provides the possibility for fraudsters to use face morphs as photographic identification in the real world. More specifically, at the point of issuing identification documents, morphs that fail to be detected will be incorporated into FOG documents for later use.

However, even if morphs are incorrectly accepted by viewers (e.g. passport issuing officers) as unaltered photographs, this does not mean that these images sufficiently resemble one or both original identities (i.e. the two faces used to create the morph). In order for fraudsters to take advantage of this method of deception at the point of document use, observers (e.g. border control officers) must accept morphs as believable photographs of those people presenting them.

One previous study has investigated face matching performance with morphs (Robertson et al., 2017) but used a computerized version of the task and only investigated morph acceptance for one of the two people pictured in each morph image (i.e. morph AB was compared to person A but not person B). In addition, as discussed earlier, those morphs were of a lower-quality in terms of realism than the ones used in the current work (see Fig. 1 for comparison between face sets). In this experiment, we investigated whether our high-quality face morphs provided acceptable identification photographs for use by both of the original people featured in the morph. In addition, we utilized a live face matching context rather than a computerized task in order to better understand the everyday process whereby people present their photo-ID documents for consideration.

## Method

### Participants

For clarity in this experiment, we labelled our participants as either models or judges. Models were those individuals who appeared in the images used and subsequently presented these images and collected responses. Judges, in contrast, were those who viewed the images and gave their responses, providing the current data for analysis.

#### Models

Forty-eight undergraduate students acted as models for this experiment (40 women, 44 self-reported as White; age *M* = 19.6 years, *SD* = 1.3 years).

#### Judges

We recruited a sample of 1410 people (985 women; age *M* = 21.8 years, *SD* = 7.1 years; 97.1% of judges self-reported as White).

All models and judges were members of a UK university. Models participated as part of their undergraduate research skills course, while judges represented an opportunity sample of students and staff that were present on campus at the time of data collection. Judges were strangers and did not know the models before recruitment. In addition, no judges had previously participated in Experiments 1 or 2. Finally, all judges confirmed that they had not already taken part in this experiment before responding.

### Materials

Digital face photographs were taken of the models. All images were constrained to reflect neutral expression, eyes on the camera, consistent posture, distance to the camera, no glasses, and hair back. A headband was provided where necessary. There were small differences in lighting conditions across subsets of models (see Fig. 1, bottom row). In addition, unlike the images used in Experiment 1, we were unable to restrict the use of visible jewelry and make-up.

We formed 20 same-sex pairs from 40 of our White models based on general descriptors (e.g. blonde hair), with this pairing carried out by two of the authors. The person each model was paired with is referred to as their “foil,” with paired individuals serving as each other’s foils. Due to the limited size of our model sample, we failed to form acceptable pairings for four White individuals. These four, along with the four non-White models, were paired with images taken from our Set 1 database, which provided a larger selection of individuals of both sexes and multiple ethnicities to choose from.

Morphs were created as in Experiment 1 (see Additional file 1), again using JPsychomorph and Adobe Photoshop in order to produce convincing images. As before, both morphs and exemplar images were given a uniform, gray background and cropped to 440 × 570 pixels. Next, any visible jewelry that appeared in the images was removed using Adobe Photoshop, primarily with the “clone stamp” tool. Finally, images were printed and laminated, with each image measuring 3.5 × 4.5 cm (see Fig. 1, bottom row for examples).

Models were provided with three images for data collection, representing the three experimental conditions: (1) match – an image of themselves; (2) morph – a computer-generated average of the model and their foil; and (3) mismatch – an image of their foil.

### Procedure

We followed the general procedure used in previous live face matching research (Ritchie, Mireku, & Kramer, 2019). The models approached people on campus and stood at a conversational distance. Each judge was shown an image corresponding to only one of the three conditions (match, morph, mismatch) and was asked, “Is the image a photo of me?” Judges had an unlimited amount of time to respond to all questions. After their yes/no response was written down, judges were then asked, “Do you have any reason why you wouldn’t accept this as an ID photo?” This open-ended question allowed judges to provide their own reasons as to why the image might not be suitable for use as identification. (Judges also had the option of giving no reason.) After these responses were written down, judges were given a morph fraud/detection tips sheet – a printed/laminated version of the tips screen used in Experiments 1 and 2. Finally, after receiving this information regarding morphs, judges were asked, “Do you think that this image is a morph?” Their yes/no responses were written down and demographic information was also collected. No feedback was given at any point during the experiment.

Each judge gave their responses to the three questions based upon only one image/condition. The image they saw was determined by cycling through all three conditions in order and then repeating this process. Each model collected responses from 30 judges (ten in each condition). However, one model was unable to collect data. As such, the final size of our dataset was 1410 judges (47 models × 30 judges). Data collection spanned approximately two weeks during the semester, with the ID photos having been taken 3–5 weeks earlier.

## Results

### Accuracy with live matching

Each model collected responses in all conditions, and following the method used previously in live face matching tasks (Kemp et al., 1997; Ritchie et al., 2019), we calculated mean accuracy (percentage correct) for the match and mismatch conditions separately (i.e. for the ten responses collected per condition) for each model. We found that accuracies in the match (*M* = 83.2%) and mismatch (*M* = 84.0%) conditions, derived from the data presented in Table 2, were comparable with overall accuracies reported in previous studies of live face matching (67.4% – Kemp et al., 1997; 83.1% – Megreya & Burton, 2008; 79.9% – Ritchie et al., 2019).^4^ As such, we can be confident that the images and foils used here did not result in overly easy or difficult matching.

**Table 2:** A summary of the data for Experiment 3. These values represent the mean proportion of “yes” responses received by models for each condition

Question	Match	Morph	Mismatch
Is the image a photo of me?	0.83 [0.78, 0.88]	0.49 [0.41, 0.57]	0.16 [0.11, 0.21]
Do you think that this image is a morph?	0.36 [0.30, 0.41]	0.64 [0.58, 0.70]	0.44 [0.37, 0.51]

*Note.* Values represent the means, with 95% confidence intervals shown in square brackets

### Are morphs accepted as ID photos?

For each model, we calculated the proportion of judges that answered “yes” for each condition when asked, “Is the image a photo of me?” These proportions were analyzed using a repeated measures ANOVA (with Condition as a within-models factor). We found a significant main effect, *F*(2, 92) = 160.91, *p* < 0.001, *η*^2^_p_ = 0.78, demonstrating that judges responded differently for the three types of image. Pairwise comparisons (Dunn-Šidák corrected) showed that judges responded “yes” significantly differently across all three conditions (all *p* values < 0.001; see Table 2).

Considering these proportions, we found that, on average, judges accepted morphs on approximately half of the times that they were presented (*M* = 0.49). However, there were substantial differences across models (proportion range: 0–1), demonstrating that the utility of a given morph depended upon the particular morph and the model attempting to use it.

**Fig. 3:**
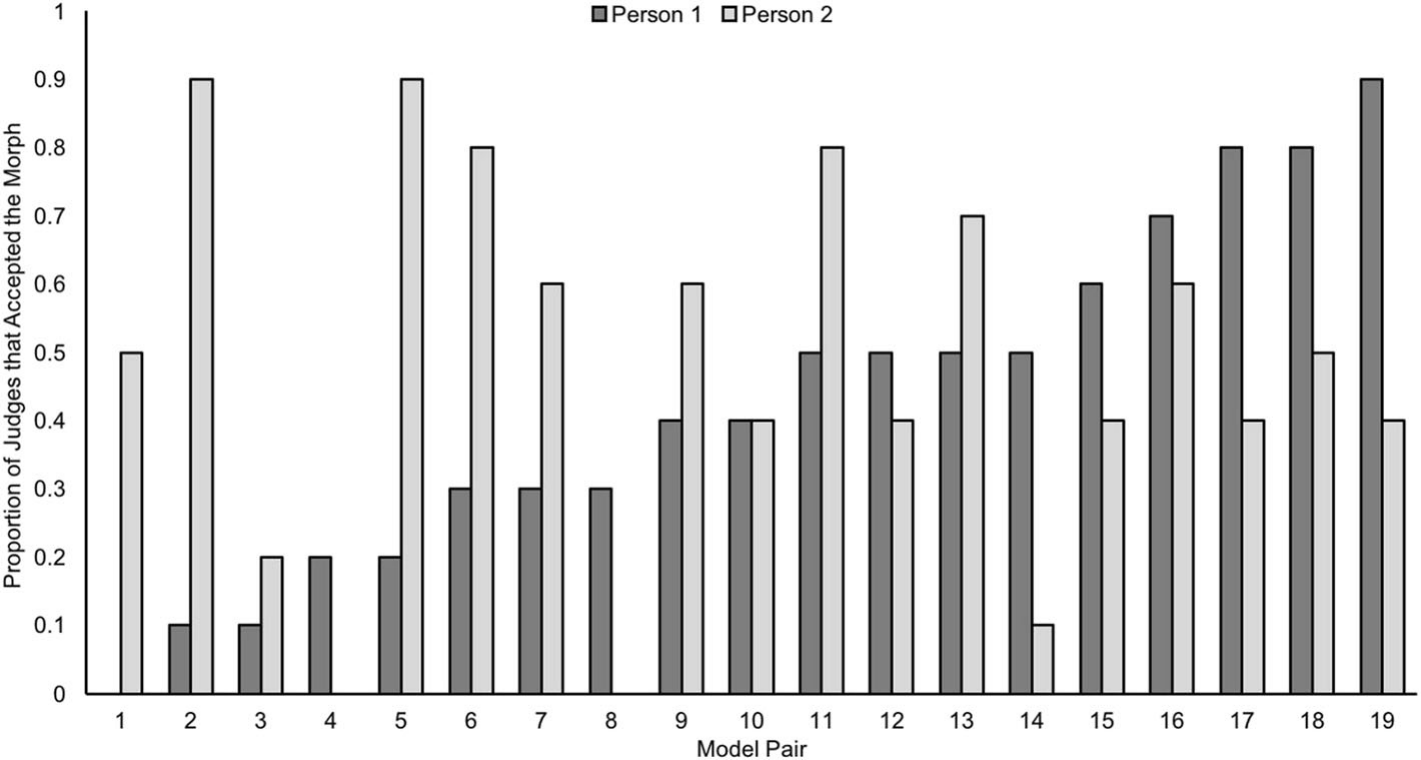
Morph acceptance for 19 of the pairs in Experiment 3. Both models within each pair are shown, illustrating the acceptance of morph images when presented by both the models they comprised

Thirty-eight of our White models formed pairs in which both individuals were used to create the morph and both collected data using that same image. With these pairs, we were able to investigate whether (different) judges accepted the same morph image when presented by each of our paired models (i.e. the AB morph presented by both person A and person B). Figure 3 illustrates these results, highlighting that for the majority of pairs, the morph was accepted as an ID image of one model noticeably more than the other. This suggests that the morphs we used, although created by equally weighting both individual images, did not resemble each individual equally.

This sheds further light on previously reported results whereby 50/50 morph images were accepted in a computerized face matching task on 68% of trials (Robertson et al., 2017). In that study, morph images were only ever presented with one of the two identities comprising the morph. Therefore, the acceptance rates when matching each of the two identities with the morph image were not investigated. Here, we show the importance of presenting the morph with both of the contributing faces, as only one of our 19 pairs produced equal morph acceptance rates for each person (pair 10 in Fig. 3).

### Are morphs detected spontaneously?

We explored the responses given when judges were asked, “Do you have any reason why you wouldn’t accept this as an ID photo?” Of the 1410 judges, only 18 gave reasons that specifically included mention of computer manipulation or similar, e.g. “doesn’t look real,” “looks filtered,” “looks photoshopped,” “avatar-like.” Of these, 15 judges took part in the morph condition (where a total of 470 judges viewed morphs).

Fourteen additional judges in the morph condition gave reasons that were less specific but suggested they had an issue with the appearance of the face, e.g. “face looks strange,” “hair line is strange,” “looks odd,” The remaining reasons given by judges for not accepting the photos either involved general issues with lighting, that the model was displaying a smile, the background color was inappropriate, or that the model was wearing a headband. These judges were approximately evenly distributed across all three conditions. The remaining judges either gave no reason or mentioned not accepting the image based on it not sufficiently resembling the model presenting it.

### Are morphs detected after instruction?

After receiving the morph fraud/detection tips sheet, judges were asked, “Do you think that this image is a morph?” For each model, we calculated the proportion of judges that answered “yes” for each condition and analyzed these using a repeated measures ANOVA (with Condition as a within-models factor). We found a significant main effect, *F*(2, 92) = 29.29, *p* < 0.001, *η*^2^_p_ = 0.39, demonstrating that judges responded differently for the three types of image. Pairwise comparisons (Dunn-Šidák corrected) showed that judges responded “yes” significantly more often (both *p*s < 0.001) to the morph than to the match and mismatch images (see Table 2). The latter two conditions did not significantly differ from each other (*p* = 0.096).

By comparing these proportions to response levels predicted by simple guessing (0.5), we found that judges were significantly better than chance at detecting that the morphs were morphs, *t*(46) = 4.92, *p* < 0.001, Cohen’s *d* = 0.72 and that the match images were not morphs, *t*(46) = 4.97, *p* < 0.001, Cohen’s *d* = 0.72. Interestingly, judges were not significantly different from chance performance for the mismatch condition, *t*(46) = 1.80, *p* = 0.079, Cohen’s *d* = 0.26.

We also calculated sensitivity measures for this question, considering the 30 judges approached by each model, using the following definitions: *Hit* – the image was a morph and judges responded “morph”; and *False alarm* – the image was an individual and judges responded “morph.” Across all models, we found that *d’* was low, *M* = 0.73, 95% confidence interval (CI) [0.53, 0.94], and there was no evidence of a response bias with regard to *c*, *M* = − 0.05, 95% CI [− 0.18, 0.08].

## Discussion

The results of this experiment demonstrated that the acceptance of morph images as ID photographs was highly image-dependent, revealing significant variation in their success as fraudulent images. In addition, we found that morph images typically resembled one of the individuals featured in the image more than the other (see Fig. 3), again resulting in large variation in their success. It is unclear as to why this was the case, giving rise to an interesting theoretical question: what causes a 50/50 morph image to equally resemble both individuals?

Previous research has shown that 50/50 morphs were judged to better resemble the more distinctive of the two individuals that were used to produce the morph (Tanaka, Giles, Kremen, & Simon, 1998). In order to test this idea, we collected ratings of distinctiveness for the images of our 38 White models that formed the pairs in Fig. 3. We found evidence suggesting that judges accepted the morph as a photo of the model more often for the more distinctive model in the pair, *r*(17) = 0.26, *p* = 0.284 (see Experiment 3b in Additional file 1 for details). The small number of pairs in the current experiment likely explains the non-significant effect and prevents our drawing any strong conclusions, although the moderate association is certainly in line with previous work (Tanaka et al., 1998). Further research might investigate this relationship in more detail.

Finally, our results suggested that judges failed to spontaneously notice that the morph images were indeed morphs (49% acceptance; see Table 2), although after receiving information/instruction regarding these types of images, detection was at levels above chance performance but was still relatively low. It is worth noting that *d’* in the current experiment (0.73) was somewhat higher than the sensitivities found in Experiments 1 and 2 (approximately 0.1–0.4). This may be due to the length of time that judges spent studying the images or models’ faces while interacting with the models (in comparison with those presented onscreen in the computer tasks), or that the morph fraud/detection tips sheet could be consulted while studying the images (versus the onscreen images being presented after the tips screen). The suggestion that detection may be higher in a live matching context is an interesting one and may have important implications for real-world procedures if supported by further research.

In this and other live matching tasks, it might be the case that participants feel more suspicious than in typical psychology experiments. When presented with a single image and asked if it depicts the person in front of them, participants may scrutinize the image more than during a multiple-trial computer task, for instance. Of course, these suspicions should be equally evident across conditions and would be apparent in their calculated response biases. However, this consideration is worth noting when designing such tasks.

## Experiment 4: Morph detection using computational modeling

So far, we have shown that naïve participants were poor at detecting high-quality morph images and that providing training or tips aimed at improving performance resulted in little or no benefit. In addition, we found that morphs were accepted as ID photos often enough that they may be feasible as tools for committing fraud.

If people are unable to detect relatively sophisticated morphs at levels that are useful for real-world fraud prevention, then perhaps computer software may represent a better approach. Recent work has begun to investigate this idea, typically utilizing state of the art computer models (e.g. deep convolutional neural networks – Raghavendra et al., 2017b). In this final experiment, we implemented a simple computational model using minimal assumptions and standard image analysis techniques. As such, we aimed to explore how a generic simulation performed with our images, allowing us to determine whether a basic computer model might outperform human morph detection.

## Method

### Materials

The images created for the above experiments were used as training and test sets here. We combined the images from the morph detection (60 individuals, 60 morphs) and training tasks (20 individuals, 20 morphs) from Experiment 1. To these, we added a subset of images used in Experiment 3 (16 individuals, 16 morphs), allowing the maximum number of images from this experiment while avoiding the use of images with overlapping identities, i.e. no individuals that formed morphs and no morphs that included individuals. This resulted in a total of 96 individuals and 96 morphs, where no identities appeared in both sets.

All images were cropped to 190 pixels wide × 285 pixels high by removing some of the background in order to be compatible with InterFace processing software (Kramer, Jenkins, & Burton, 2017).

### Model

We used linear discriminant analysis (LDA) to train our model to group different images of the same type (individuals, morphs) together. This technique minimizes intra-class differences while maximizing inter-class differences and has been used previously to simulate familiar face recognition (Kramer, Young, & Burton, 2018; Kramer, Young, Day, & Burton, 2017). When classifying images, it is common to have fewer sample vectors (images) than features (pixels). In such cases, LDA cannot be carried out without first reducing the number of feature dimensions. One popular approach is to initially subject the faces to principal components analysis (PCA), resulting in a lower-dimensional description of “eigenfaces,” which represent the variability in the image set (e.g. Bekios-Calfa, Buenaposada, & Baumela, 2011). Here, we adopted this PCA-based approach to dimension reduction.

All images were shape-standardized by morphing them to a template derived from the average shape of the training set (Burton, Miller, Bruce, Hancock, & Henderson, 2001; Craw, 1995). This standardization was based on the alignment of 82 fiducial points for each image (e.g. corners of eyes, corners of mouth, etc.; for technical details, see Burton, Kramer, Ritchie, & Jenkins, 2016, and for downloadable face processing software, see Kramer, Jenkins, & Burton, 2017). Assignment of these fiducial points was carried out using a standard semi-automatic process requiring just five manually entered landmarks (see Kramer, Young, et al., 2017, for details). PCA was then computed on these normalized images.

In order to reduce the number of dimensions describing the resulting space without significant loss of variability, we retained the highest 67 components only (which explained approximately 95% of the variance in the image RGB information). The images’ projections on these principal components were then entered into an LDA, where the two classes represented individuals and morphs. The result is a reshaped space comprising one dimension (the number of classes minus 1).

### Procedure

On each iteration of our simulation, we randomly selected 86 individuals and 86 morphs to represent the training set. The remaining 10 individuals and 10 morphs (approximately 10% of the total) were used as novel, test images. We then ran the PCA + LDA procedure without these test photos (i.e. with 172 training images). Next, we projected each untrained test image into the resulting one-dimensional space.

We calculated the mean location along this dimension of the 86 training individuals and, separately, the mean location of the 86 training morphs. These two values were used when classifying untrained images – each novel image was labelled according to which of the two points it fell closest to on the dimension. Finally, we calculated percentage correct, along with *d’* as before: *Hit* – the image was a morph and was categorized as “morph” by the model; and *False alarm* – the image was an individual and was categorized as “morph” by the model.

We ran 100 iterations of the simulation, each time randomly selecting images for the training and test sets. For each iteration, percentage correct and *d’* values were calculated.

## Results

Over 100 iterations, the performance of the model was as follows: percentage correct, *M* = 68.4%, 95% CI [66.0, 70.9]; and *d’*, *M* = 1.01, 95% CI [0.88, 1.15]. Although imperfect, these values were higher than those found in the previous experiments with human morph detection (see Tables 1 and 2, and Additional file 1: Table S1). Indeed, a direct comparison of the *d’* sensitivities for the best human performance (in Experiment 3) and those found here showed a significant computer advantage, *t*(145) = 2.28, *p* = 0.024, Cohen’s *d* = 0.40.

## Discussion

**Fig. 4:**
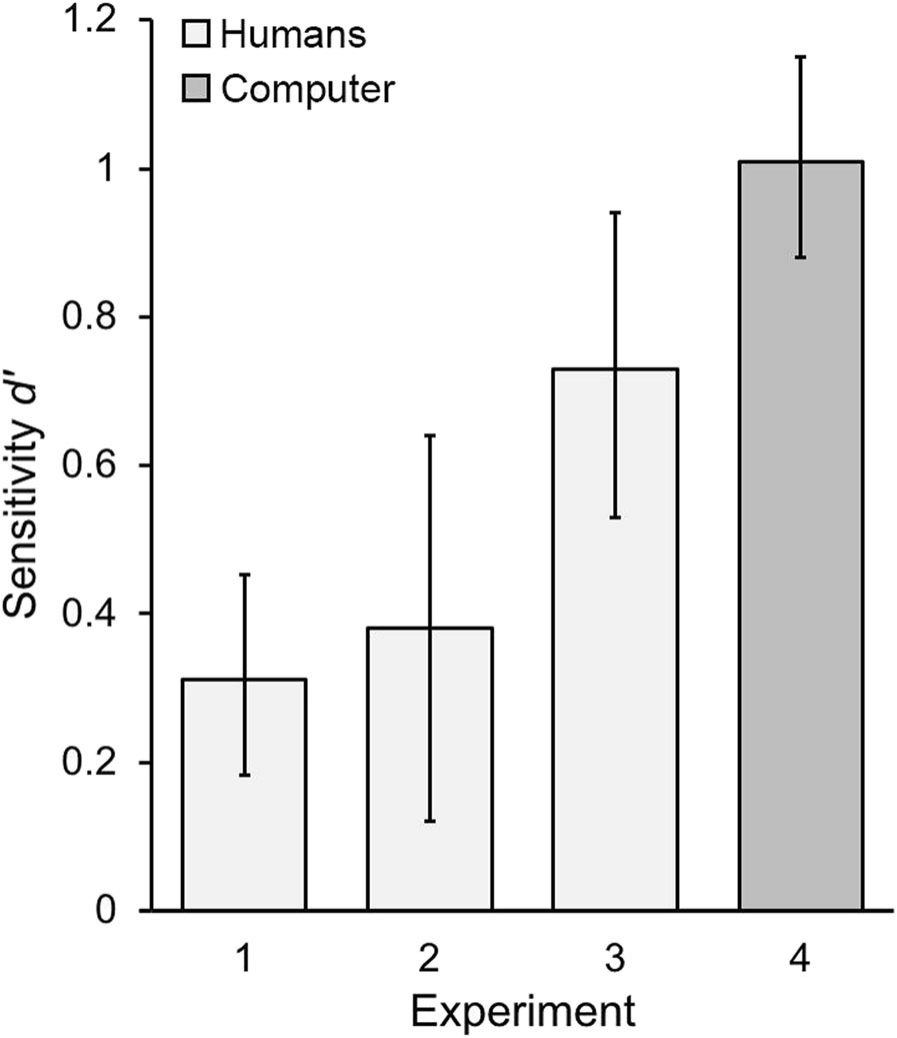
A comparison of all four experiments. A plot of sensitivity data for the human detection of morphs for conditions where the morph detection tips were provided beforehand (*light gray*), along with the computer model’s performance (*dark gray*). *Error bars* represent 95% confidence intervals

The results of this experiment demonstrated that a basic computer model outperformed humans with regard to morph detection. Given that the model was provided with training images to “learn” from, we might consider this to be comparable with our participants in Experiments 1–3 who received morph training and/or tips regarding how to detect morph images. That the model showed higher sensitivity to the detection of morphs (see Fig. 4) suggests that computational techniques might be better suited to this task, where image artefacts indicative of morph creation (e.g. impossible lighting and reflectance) may be present but imperceptible when viewed by people. This supports the growing body of research investigating the use of computational techniques in morph detection (e.g. Seibold, Hilsmann, et al., 2018).

The current model was trained and tested using images with varying characteristics. The photos of individuals (and hence the resulting morphs) showed significant differences in brightness and other qualities across the image sets used; this may have resulted in a more robust “morph detector” since the model was trained to classify images irrespective of these irrelevant sources of variation. One might predict that further increases in this type of variation by including morphs created from many different photo sets would result in additional performance improvements.

That a simple model, based on a PCA + LDA process and using only RGB pixel values, performed with more success than our participants is revealing. This demonstrates that the necessary information was present in the images in order for detection to take place, but computers were better suited to making use of this information. Here, we explored a basic “proof of concept” model with our images, while more advanced computational techniques are beginning to demonstrate impressively high levels of detection using a variety of approaches (e.g. Seibold, Samek, et al., 2018).

### General discussion

In the current set of experiments, we investigated human performance in morph acceptance and detection tasks. Although recent work has provided some initial insights into this field (Robertson et al., 2017, 2018), we have argued that the use of unsophisticated morph images failed to quantify the viability of real-world morphs in everyday fraud. Here, we have demonstrated that people were poor when asked to detect high-quality morphs and that training paradigms of the type previously implemented did not produce significant improvements (Experiments 1 and 2). In a live matching scenario, morphs were often accepted as ID images and, in many instances, judges failed to notice that the morph images were indeed morphs, either spontaneously or after receiving tips/instruction (Experiment 3). Finally, a relatively basic computational model was able to detect our morphs at levels higher than those found with our human participants, suggesting a more suitable alternative for fraud prevention (Experiment 4).

Previous work suggested that morph detection was difficult, but that providing a tips screen with an example morph, along with suggestions for how to identify them, resulted in significant improvements (Robertson et al., 2018). In contrast, performance with our higher-quality morphs found even lower levels of detection and no benefit from tips or training (see Fig. 2). Importantly, the tips we provided were specifically tailored to our morphs in order to give participants the opportunity to gain useful knowledge that might help with detection. It is worth noting that, had we used the tips provided by the previous researchers, participants would have been searching for artefacts and signs that were never present. In reality, as morphs become increasingly sophisticated, there will inevitably come a point at which there are no visible signs (at least, to humans) betraying the image as a morph. Indeed, we may already be reaching that point with our images, given the lack of a tips/training benefit. Therefore, it seems reasonable to assume that no method of training will provide human viewers with the ability to detect morphs currently or, at the very least, in the near future.

Using a forced choice task with chance performance at 50%, we found that morph detection was no better than this level. This result highlights the problematic nature of detecting this type of fraud for professionals when issuing documents or making comparisons with morph images. Although Experiment 2 provided limited evidence that a tips screen improved performance, such increases were very small and the resulting sensitivity (*d’* = 0.38, the highest found across Experiments 1 and 2) could not be considered useful or effective if applied to a national security context.

Importantly, for face morphing attacks to be of significant risk to security, detection is only half of the story. Morph images must also look sufficiently like the person using the document in order for them to be of practical value – a morph that looks like a real photo but does not resemble the person carrying it will pose no threat. Previous work suggested that 50/50 morphs were accepted as depicting the same person as the comparison image on 68% of trials (Robertson et al., 2017). Here, in a live matching scenario, we found that our higher-quality morphs were accepted on 49% of occasions. We suggest several possibilities for why our morphs were seemingly less successful. First, Robertson et al. (2017) selected identities from a substantially larger initial set, which may have resulted in pairs of faces that more closely resembled each other. Here, as best we could, we limited ourselves to forming pairs from only 48 identities. Second, live face matching (used here) may allow judges to access additional information regarding face variability, provided by the brief initial conversation with the models (mirroring the informal interviews conducted by border control officers). In contrast, comparison with a second photograph (Robertson et al., 2017) will be inherently more limiting in the information it conveys. Third, participants in a 49-trial computer task (Robertson et al., 2017) may be less conscientious in their responses. Here, each judge only considered one image/comparison and so we might predict that more thought went into their decision. Fourth, it appears that Robertson et al. only investigated acceptance rates for one identity from each pairing. If their sample of identities happened to consist of those that better resembled the morphs they were contained within (in comparison with the second identities not featured) then this would result in inflated estimates. In Experiment 3, we provided strong evidence that in almost every pair, the morph resembled one identity more than the other (see Fig. 3). If we only considered responses for the identity in each pair that saw higher acceptance rates when asked if the image was a photo of the model, our mean level would increase to 62%.

Interestingly, we found that accuracy in detecting morphs was notably higher in the live matching context in comparison with the two computer tasks (see Fig. 4). This may be due to the lower-quality of the morphs used, given that lighting conditions were not controlled when photographing the individuals in Experiment 3. As such, the resulting morphs may have looked more artificial when presented with dark/light artefacts (see Fig. 1, bottom row). In addition, and as mentioned above, judges were exposed to information regarding face variability that is absent in photographs. At the point of presentation, border control officers purposely converse with travelers while checking their documents. Although this may be primarily to observe any suspicious behaviors, the process also provides additional face information. Therefore, when attempting to detect morphs in a face matching situation, live comparisons better represent the typical context in which professionals find themselves and are also ones in which performance levels may be optimal (although still error-prone).

Perhaps surprisingly, we found that morphs typically resembled one identity contained within them more than the other, as implied by acceptance rates for the two models presenting each morph image. At first consideration, such patterns might be predicted by categorical perception studies, where presenting a continuum of images morphing from one face to another will result in a perceptual boundary – either side of this boundary, each of the identities is perceived despite the continuous nature of the images (e.g. Beale & Keil, 1995). However, this result may be limited to familiar faces or those that have been explicitly categorized beforehand (Kikutani, Roberson, & Hanley, 2010). Importantly, when presenting a morph to a stranger, no prior knowledge of either identity exists. Instead, they must simply decide whether the morph sufficiently resembles the photo/live face or not. That one identity clearly “dominates” within the morph image is a finding predicted by previous work demonstrating that a 50/50 morph better resembled the more distinctive identity (Tanaka et al., 1998). Indeed, some preliminary data (see Experiment 3b in Additional file 1) support this idea.

Our human performance results demonstrate that there are many instances in which morphs could prove viable for fraudsters, primarily because people were poor at detecting morph images and training provided little or no benefit. Much of the research on face morphing attacks has been focused on computational methods of detection (e.g. Seibold, Hilsmann, et al., 2018), with the implication that developing effective software is the preferred method of tackling this issue. Here, we showed that even a simple computational model, based upon PCA and LDA, was more successful than our human participants in detecting morphs. As these techniques become increasingly advanced (e.g. deep convolutional neural networks – Raghavendra et al., 2017b), we predict that success rates will supersede human abilities. Problematically, at least in many security contexts, final decisions are often made by human operators, and as a result, face morphing attacks may go undetected. As Ferrara et al. (2014) note, the best solution is for government officials to directly acquire ID photos at the place of issue, preventing fraudsters from submitting pre-made morph images for consideration. However, this does not prevent other methods of fraud from taking place (e.g. the use of hyper-realistic silicone masks; Sanders et al., 2017).

## Conclusions

Across four experiments, we have investigated human and computer performance with high-quality face morphs. Our results show that morph detection is highly error-prone and that training does not provide a useful solution to this problem. Instead, computer algorithms may be a better method for minimizing the frequency with which face morphing attacks are missed. Interestingly, morphs typically resemble one individual in the pair to a greater extent than the other, suggesting a possible limitation for fraudsters who plan to use such techniques. The results of these experiments have important implications for real-world national security measures and highlight that it is essential for researchers to consider the quality of morphs that are likely to be employed by fraudsters in real-world settings.

## Additional file

Additional file 1: Additional information regarding image creation and analysis, as well as Experiment 3b investigating distinctiveness. (DOCX 28 kb)

## Data Availability

The datasets used and analyzed during the current study are available from the corresponding author on reasonable request.

## Abbreviation

FOG: Fraudulently obtained genuine

## Footnotes

1 We have presented images from Experiment 3 as we did not have permission to publish the images used in Experiments 1 and 2.

2 We chose to use this wording rather than “Which of these images have been digitally manipulated?” (Robertson et al., 2018) because we felt that this was a clearer instruction. Importantly, all our images and those used by Robertson and colleagues were digitally manipulated in that all had been cropped, placed on a uniform background, etc. Therefore, our rewording makes it specifically applicable to the detection of morphs and not these other manipulations.

3 We were unsure as to whether participants would realize, based on the wording of the question, that they could enter between zero and ten letters as their response. However, a pilot study of the task with a different sample of five people revealed a response range of 0–5 letters per trial. As such, we were confident that participants would not misinterpret the question.

4 After excluding all non-White models and judges, these accuracies remained virtually unchanged: match *M* = 83.7%, mismatch *M* = 83.9%.
