# Supplementary material for: Face morphing attacks: Investigating detection with humans and computers
Source: Cogn Res Princ Implic. 2019 Jul 29;4:28. doi: 10.1186/s41235-019-0181-4 (PMC6663958; doi:10.1186/s41235-019-0181-4)
Supplement: Supplementary file 1 — Additional information regarding image creation and analysis, as well as Experiment 3b investigating distinctiveness. (DOCX 28 kb) [file 41235_2019_181_MOESM1_ESM.docx]

**Face Morphing Attacks: Investigating Detection with Humans and Computers**

**Additional file 1**

**Experiments 1 and 2: Image creation**

All images were constrained to reflect neutral expression, eyes on the camera; consistent posture, lighting, and distance to the camera; no glasses, jewellery, or make-up; and hair back. Due to the fact that lighting conditions differed across the two sets (one set of faces appeared lighter than the other), we did not mix images from both sets in any given task to avoid participants incorrectly believing that this visual difference could be used when identifying individuals versus morphs. We also therefore only formed ‘within set’ pairs of individuals when creating our morph images to avoid averaging across different lighting conditions.

To create morphs for our pre- and post-training morph detection tasks, we paired 120 individuals from our Set 1 database. We included only individuals who self-reported as White, and formed same-sex pairs based on general descriptors (e.g., blonde hair). In order to produce 60 morphs (43 female), we first manually delineated each photograph using JPsychomorph software, fitting a 192-point custom template. This software was then used to create 50/50 morphs of the image pairs (i.e., equally weighting both faces), processing the textures using wavelet MRF (Markov Random Field; Tiddeman, Stirrat, & Perrett, 2005) to increase their perceived realism. Next, the morphs were modified using Adobe Photoshop CC 2018 software in order to remove any noticeable artefacts of the averaging process (e.g., the presence of a secondary outline for the hair or glitches around the neck and shoulders). Here, we used only 50/50 morphs, in contrast with Robertson et al.’s (2018) use of different numbers of 30%, 40% and 50% morphs. Finally, images were given a uniform, grey background and cropped to 440 x 570 pixels (see Fig. 1 bottom row).

For our exemplar (non-morphed) images shown in these two tasks, we selected 60 White faces (33 female) from Set 1. Importantly, none of the individuals chosen had appeared in any of the morph pairings, meaning that throughout the experiment, each individual appeared only once. In order to standardise the exemplars with respect to our morphs, all images were given the same grey backgrounds and cropped to the same dimensions.

**Experiment 1: Signal detection analyses**

Following Robertson et al. (2018), we analysed the data using signal detection measures by calculating sensitivity indices (*d’*) and criterion values (*c*). Robertson et al. (2018) did not provide details of how these measures were calculated and so our interpretation was to calculate hits and false alarms with each out of a maximum of 30 (incorporating all ‘morph detection’ faces for a given task).

We used the following definitions: *Hit* – the image was a morph and participants responded “morph”; and *False alarm* – the image was an exemplar and participants responded “morph”. For *d’*, we found a significant main effect of Group, *F*(1, 78) = 5.26, *p* = .025, *η*^2^_p_ = 0.63, with the control group (*M* = 0.33) performing better than the training group (*M* = 0.12). Mirroring the previous analysis, this group difference translates into the control group scoring an average of only one more hit and one fewer false alarm on the task than the training group. As with the percentage correct analysis, we found no main effect of Session, *F*(1, 78) = 0.25, *p* = .620, *η*^2^_p_ < 0.001, and no significant Group x Session interaction, *F*(1, 78) = 0.00, *p* = .994, *η*^2^_p_ < 0.001.

For *c*, we found no main effect of Group, *F*(1, 78) = 0.75, *p* = .389, *η*^2^_p_ = 0.10, or Session, *F*(1, 78) = 0.38, *p* = .538, *η*^2^_p_ = 0.01. However, there was a significant Group x Session interaction, *F*(1, 78) = 4.64, *p* = .034, *η*^2^_p_ = 0.06. We therefore considered the simple main effects of Session at each level of Group. These simple main effects were significant for the training group, *F*(1, 78) = 3.84, *p* = .054, *η*^2^_p_ = 0.05, but not for the control group, *F*(1, 78) = 1.18, *p* = .281, *η*^2^_p_ = 0.01. As such, the morph training task (but not the control task) produced a change in participants’ response criteria when completing the morph detection task for the second time, resulting in their responding ‘morph’ more often in comparison with the first time they completed the task.

For a summary of the results for Experiment 1, including the percentage correct values analysed in the main text, see Table S1.

Table S1

A summary of the data for Experiment 1

| Group | Session | Percentage Correct, % | Hits | False Alarms | *d’* | *c* |
| --- | --- | --- | --- | --- | --- | --- |
| Morph Training | Pre-training | 52.3 [49.2, 55.3] | 0.38 [0.33, 0.42] | 0.33 [0.29, 0.38] | 0.14 [-0.04, 0.32] | 0.41 [0.31, 0.51] |
| Morph Training | Post-training | 52.0 [49.6, 54.3] | 0.41 [0.36, 0.45] | 0.37 [0.33, 0.40] | 0.11 [-0.03, 0.24] | 0.31 [0.20, 0.41] |
| Control | Pre-training | 56.1 [53.0, 59.1] | 0.42 [0.38, 0.47] | 0.30 [0.26, 0.35] | 0.35 [0.17, 0.52] | 0.38 [0.28, 0.48] |
| Control | Post-training | 55.9 [53.6, 58.2] | 0.40 [0.36, 0.45] | 0.29 [0.25, 0.32] | 0.31 [0.18, 0.45] | 0.44 [0.34, 0.55] |

*Note.* Values represent the means, with 95% confidence intervals shown in square brackets.

**Experiment 3b: Distinctiveness ratings**

In Experiment 3, we found that morph images typically resembled one of the individuals featured in the image more than the other (see Fig. 3). Previous research has shown that 50/50 morphs were judged to better resemble the more distinctive of the two individuals that were used to produce the morph (Tanaka, Giles, Kremen, & Simon, 1998). In order to test this idea, we collected ratings of distinctiveness for our models.

**Method**

***Participants***

A sample of 30 American volunteers (14 women; age M = 35.7 years, SD = 10.4 years; 90% self-reported as White) gave informed, onscreen consent before participating in the experiment and were provided with an onscreen debriefing upon completion. Participants were recruited through Amazon Mechanical Turk. There was no overlap between this sample and those who participated in Experiments 1, 2, and 3.

***Stimuli***

The images of our 38 White models whose live face matching results were reported in Fig. 3.

***Procedure***

The experiment was completed online using the Qualtrics survey platform (www.qualtrics.com). After consent was obtained, participants provided demographic information (age, sex, and ethnicity).

Each participant was asked to rate all 38 images, presented in a random order, for distinctiveness. Following Leder and Bruce (1998), the instruction presented onscreen throughout the experiment read, “How distinctive would you rate this face? (A distinctive face is one that would stand out in a crowd.)” Responses were given using a 1 (not distinctive) to 9 (very distinctive) Likert scale.

**Results**

Cronbach’s α for interrater reliability was .84. Distinctiveness ratings were averaged across participants to provide a value for each image. For the 19 pairs of models, we than calculated the difference between the distinctiveness values of the two models (Model A minus Model B). Similarly, we calculated the difference between the proportions representing how often the morph was accepted as each of the two models, as shown in Fig. 3 (again, Model A minus Model B). Finally, we correlated these two sets of difference values.

If Model A was rated as appearing more distinctive than Model B, we should predict that the morph would be accepted more often for Model A than Model B, resulting in a positive correlation. Our analysis produced a moderate, but nonsignificant, association, *r*(17) = .26, *p* = .284. This finding is in line with previous work (Tanaka et al., 1998), although our small number of pairs explains the lack of a statistically significant result.

**References**

Leder, H., & Bruce, V. (1998). Local and relational aspects of face distinctiveness. *The Quarterly Journal of Experimental Psychology: Section A, 51*(3), 449-473.

Robertson, D. J., Mungall, A., Watson, D. G., Wade, K. A., Nightingale, S. J., & Butler, S. (2018). Detecting morphed passport photos: A training and individual differences approach. *Cognitive Research: Principles and Implications, 3*(27), 1-11.

Tanaka, J., Giles, M., Kremen, S., & Simon, V. (1998). Mapping attractor fields in face space: the atypicality bias in face recognition. *Cognition, 68*(3), 199-220.

Tiddeman, B. P., Stirrat, M. R., & Perrett, D. I. (2005). Towards realism in facial image transformation: Results of a wavelet MRF method. *Computer Graphics Forum, 24*(3), 449-456.
